# Practical therapeutic approach in the management of diabetes mellitus secondary to Cushing’s syndrome, acromegaly and neuroendocrine tumours

**DOI:** 10.3389/fendo.2023.1248985

**Published:** 2023-09-28

**Authors:** Valentina Guarnotta, Fabrizio Emanuele, Riccardo Salzillo, Maria Bonsangue, Carlotta Amato, Mariagrazia Irene Mineo, Carla Giordano

**Affiliations:** Department of Health Promotion, Mother and Child Care, Internal Medicine and Medical Specialties, Section of Endocrinology, University of Palermo, Piazza delle Cliniche 2, Palermo, Italy

**Keywords:** counterregulatory hormones, GH secreting, glucagonoma, pheochromocytoma, cortisol, catecholamine

## Abstract

Cushing’s syndrome, acromegaly and neuroendocrine disorders are characterized by an excess of counterregulatory hormones, able to induce insulin resistance and glucose metabolism disorders at variable degrees and requiring immediate treatment, until patients are ready to undergo surgery. This review focuses on the management of diabetes mellitus in endocrine disorders related to an excess of counterregulatory hormones. Currently, the landscape of approved agents for treatment of diabetes is dynamic and is mainly patient-centred and not glycaemia-centred. In addition, personalized medicine is more and more required to provide a precise approach to the patient’s disease. For this reason, we aimed to define a practical therapeutic algorithm for management of diabetes mellitus in patients with glucagonoma, pheochromocytoma, Cushing’s syndrome and acromegaly, based on our practical experience and on the physiopathology of the specific endocrine disease taken into account. This document is addressed to all specialists who approach patients with diabetes mellitus secondary to endocrine disorders characterized by an excess of counterregulatory hormones, in order to take better care of these patients. Care and control of diabetes mellitus should be one of the primary goals in patients with an excess of counterregulatory hormones requiring immediate and aggressive treatment.

## Introduction

Diabetes mellitus (DM) is defined as a condition of hyperglycaemia resulting from a defect in insulin secretion and/or action. The most common forms of DM secondary to endocrinopathies are related to an excess of counterregulatory hormones, including glucagon, cortisol, catecholamines and GH, which are able to contrast insulin action with the development of DM.

In particular, glucagon increases endogenous glucose production through both glycogenolysis and gluconeogenesis. It acts very rapidly and is secreted in response to acute hypoglycaemia.

The effects of catecholamines on glucose counter-regulation are also very rapid and result from several mechanisms occurring at different sites. They directly stimulate glucose production, reduce peripheral sensitivity to insulin and induce lipolysis in adipose tissue.

Cortisol acts mainly in the muscles, stimulating protein catabolism and release of amino acids into the circulation, which can be subsequently used in the liver for gluconeogenesis. In addition, this hormone stimulates release of free fatty acids from adipose tissue and impairs tissue insulin action.

Finally, the GH effect in glucose counter-regulation is mainly related to reduced glucose utilization in insulin-dependent tissues. This hormone also stimulates lipolysis and protein synthesis and impairs tissue sensitivity to insulin action.

In accordance with the physiological effects of counterregulatory hormones, pathological chronic excess of any of them may cause secondary DM. Indeed, increased blood glucose is typically part of the syndromes observed in the presence of tumours producing these hormones. Although, in absolute terms, diabetes accompanying these heterogeneous syndromes is quite rare, it is important that these conditions are recognized, because hyperglycaemia may possibly be reversed by specific treatments, in particular by removal of the underlying tumour.

However, the presence of glucagonoma, pheochromocytoma, Cushing’s syndrome and acromegaly is often associated with DM, requiring immediate treatment, until patients are ready to undergo surgery. In addition, Cushing’s syndrome and acromegaly are often medically treated with somatostatin analogues which can cause hyperglycaemic effects (1). As reported by the most recent ADA guidelines, the goal of treatment of DM should be reduction of cardio-renal risk and achievement and maintenance of glycaemic and weight control (2). Managing DM, notably secondary to endocrine disorders, is not always immediate. For this reason, we aimed to define a practical therapeutic algorithm for management of diabetes mellitus in patients with glucagonoma, pheochromocytoma, Cushing’s syndrome and acromegaly.

## Treatment strategies for diabetes mellitus secondary to endocrine disorders

According to the most recent international guidelines on type 2 DM (T2DM) management, the therapy should be patient-centred and not glycaemia-centred (2). Therefore, the treatment goal for DM should be to prevent or delay diabetes-related complications and improve quality of life.

## Metformin

Metformin is generally the first approach for treatment of T2DM. It can improve glycaemic control by several different mechanisms: in the liver, by inhibiting hepatic gluconeogenesis and improving hepatic insulin sensitivity, resulting in a decrease in fasting glucose levels and, in the intestine, by increasing intestinal glucose utilization and stimulating GLP-1 secretion (3). It acts via both AMP-activated protein kinase (AMPK)-dependent and AMPK-independent mechanisms (4).

Metformin decreases glycated haemoglobin (HbA1c) and improves glycaemic control, without causing hypoglycaemia by itself (5). The effect of metformin on weight loss is almost absent, or may induce just a modest weight loss up to 4 kg (5). By contrast, it can significantly reduce triglycerides and low-density-lipoprotein cholesterol (LDL-C) levels, without altering high-density-lipoprotein cholesterol (HDL-C) levels (6), and also decrease cardiovascular risk by approximately 40% (7). Metformin improves diabetes all-cause mortality and it is safe for patients with heart failure without worsening heart failure outcomes (8). Metformin’s most common side effects are gastrointestinal ones: nausea, diarrhoea and abdominal discomfort, usually occurring in up to 50% of patients and often causing a reduction in compliance (5).

Another very important but, luckily, very rare side effect of metformin therapy is lactic acidosis (5). This is a potential lethal complication that may occur in patients with renal dysfunction, leading to a reduction in renal metformin excretion. It causes an increase in metformin blood levels and inhibition of mitochondrial functions, resulting in overproduction of lactic acid (5).

## SGLT-2 inhibitors

Gliflozins are glycosuric drugs working on sodio-glucose co-transporters (SGLT).

SGLT are located in the proximal convoluted tubule (TCP) of renal glomerulus and involved in reabsorption of glucose from the glomerular filtration rate. There are two different types of SGLT: SGLT1, responsible for reabsorption of about 10-20% of filtered glucose; and SGLT2, responsible for the remaining 80-90% glucose reabsorption (9–11). Both these transporters ensure total reabsorption of filtered glucose, unless glycaemia is over 180 mg/dl, and in this case glycosuria occurs.

Diabetic patients express SGLT2 receptors more frequently than non-diabetic ones, so glycosuria occurs when glycaemic values reach more than 220 mg/dl (12). Therefore, gliflozins reduce glycaemia by inhibiting SGLT2 transporters and excreting glucose in the urine.

Currently the only drugs approved by FDA for treating T2DM patients are empagliflozin, canagliflozin, dapagliflozin, and ertugliflozin (2).

Dapaglifozin is generally started at 5 mg and can be up-titrated to 10 mg. It is highly sensitive for SGLT-2 (13).

Canaglifozin is slightly less selective for SGLT2. It can be administered once daily before meals/a meal, at a starting dose of 100 mg with a maximum dose of 300 mg/day (14).

Empaglifozin is the SGLT2 inhibitor (SGLT2-i) with the highest selectivity for SGLT2 over SGLT1 (>2,500-fold). The starting dose is 10 mg, once daily and can be titrated up to 25 mg once daily (14). Ertuglifozin can be started at the dose of 5 mg and it can be titrated up to 15 mg once daily (15).

The most important advantage of SGLT2-i receptors is their ability to lower glucose levels, even reducing HbA1c levels by 0.4-1.1% (16). A significant reduction in HbA1c levels has been seen in every single drug (from a maximum of -0.9% for canagliflozin 300 mg, to -0.7% for empagliflozin 25 mg, down to -0.6% with dapagliflozin 5 mg) against a placebo (13). They are effective both on fasting and postprandial blood glucose levels, also preventing the risk of hypoglycaemia by stimulating glucagon secretion (17).

SGLT2-i can also cause weight loss of approximately 1-3 kg (18). Further, gliflozins have a significant effect in lowering blood pressure, as they reduce blood volume by causing osmotic diuresis (11, 18), have anti-inflammatory effects and improve hepatic steatosis (19–21).

Cardio-renal protective effects of SGLT2-i in patients with T2DM have been demonstrated in clinical trials, which showed a decrease in MACE by 10%, notably in patients with established atherosclerotic cardiovascular disease, and a decrease in hospitalization for heart failure by 30% (22–28).

With regard to kidney disease, SGLT-2i reduced the risk of kidney progression by 40% in diabetic patients (29).

There are also many disadvantages of using gliflozins. First of all, they seem to raise the rate of genital mycotic infections, including balanitis and vulvovaginitis (18). Indeed, some studies have reported that gliflozins increase the frequency of bacterial infections of the urinary tract (18). In both cases, mycotic and bacterial infections are due to glycosuria, which favours proliferation of micro-organisms.

A limit of gliflozin efficacy concerns renal function. Indeed, while these drugs have beneficial effects in reducing progression of renal disease, their glycaemic efficacy reduces as the glomerular filtration rate falls (11). Moreover, their ability to reduce blood pressure can cause hypotension, especially in people treated with drugs acting on the renin-angiotensin system (18).

There are different eGFR thresholds for SGLT2-i. It is recommended not to start therapy with canagliflozin and empaglifozin if the patient’s eGFR is less than 45 mL/min/1.73 m^2^, while the threshold to start Dapagliflozin is eGFR < 60 mL/min/1.73 m^2^ (30).

Finally, gliflozins should be used with caution because they can induce ketoacidosis. Indeed, the drastic fall of blood glucose levels reduces insulin levels and raises glucagon (9). The metabolism shifts in a catabolic sense, increasing hepatic production of ketone bodies, leading to ketoacidosis (9).

Recent studies have also shown that SGLT2-i directly stimulate pancreatic α-cells with increased glucagon secretion (31).

## DPP4 inhibitors

Dipeptidyl peptidase inhibitors (DPP4-i) are easy-to-use and well tolerated drugs (32), which exert their hypoglycaemic action by preventing degradation of GLP-1 and GIP (33), thus resulting in reduction of blood glucose and HbA1c values. They are associated with a low risk of hypoglycaemic events and are neutral regarding weight gain (34). DPP4 is considered an adipokine with a pleiotropic effect, whose levels are increased in conditions of obesity, insulin resistance and T2DM; it is also involved in lipid metabolism and in the atherosclerotic process.DPP4-i include sitagliptin, alogliptin, linagliptin and saxagliptin. These drugs do not show cardioprotective effects, but can be considered safe for cardiovascular disease (except for saxagliptin, which worsens the risk of heart failure) (35–37). Other studies have reported a nephroprotective effect of these drugs due to a decrease in proteinuria (34).

Against the good tolerability of these drugs, several studies have shown an increased risk of acute pancreatitis in patients treated with DPP4-i. Nevertheless, DPP4-i are indicated in the treatment of frail and elderly patients (32).

## GLP-1 analogues

GLP-1 (Glucagon-like peptide 1) analogues or receptor agonists (RA) are antidiabetic drugs acting on the incretin system. Their mechanism of action is based on the ‘incretin effect’, that is the phenomenon whereby oral glucose ingestion produces a greater insulin response than that induced by intravenous glucose (38). This effect is mediated by incretins, specific gastrointestinal hormones, mainly GLP-1 and GIP (Gastric inhibitory peptide). GLP-1 acts on post-prandial glycaemia, stimulating insulin production by pancreatic β cells and inhibiting glucagon secretion by pancreatic α cells (38). On the other hand, GIP stimulates both insulin and glucagon secretion.

The biggest difference between GLP-1 and GIP is that GLP-1 inhibits the sense of hunger by acting both on the hunger centres of the central nervous system and peripherally by slowing gastric emptying, thus determining a significant reduction of caloric intake and therefore of body weight, while GIP seems to be devoid of these effects.

Therefore GLP-1 RA mimic the effect of GLP-1 on its receptor, causing the whole cascade of events mentioned above. Endogenous GLP-1 is a 30 or 31 amino acid long peptide hormone, secreted by enteroendocrine L cells in the distal intestine, and alpha cells in the pancreas and the central nervous system (33). It has a very short half-life. It remains in the circulation about 1-2 minutes, before being digested by DPP4 and then excreted. A drug molecule, similar to GLP-1 but able to resist DPP4 degradation longer, in order to make its half-life longer, has been produced (39).

GLP-1 RA can be distinguished in long-acting, with weekly (Semaglutide, Dulaglutide, Exenatide) administration, and short-acting, with daily administration (Lixisenatide and Liraglutide). According to the method of administration, they can be distinguished into injectable or oral formulations (the only one existing at this time is oral Semaglutide).

The cardio-renal protective effects of GLP-1 analogues in patients with T2DM have been widely demonstrated in clinical trials (40–45).

In May 2022 the US Food and Drug Administration (FDA) approved tirzepatide as a new therapeutic strategy for T2DM (46). Tirzepatide acts simultaneously on both GLP-1 and GIP receptors (47, 48). It’s a new dual GIP/GLP-1 receptor co-agonist, that acts simultaneously on both GLP-1 and GIP receptors, also expressed in specific regions of the brain that regulate food intake (29–31). It has a mean half-life of 5 days, enabling once-a-week administration (49).

The SURPASS programme analysed the superiority or non-inferiority of tirzepatide vs. placebo or active comparator (semaglutide, insulin degludec, insulin glargine) in mean change in HbA1c, and demonstrated the superiority of tirzepatide in improving not only glycaemic control, but also weight loss in a dose-dependent way and in reducing blood pressure and LDL-C and triglycerides (50).

Adverse effects reported for tirzepatide were similar to the ones of the selective GLP-1 analogues, including nausea, vomiting, constipation and diarrhoea (50).

According to several studies, GLP-1 analogues are very effective in reducing blood sugar and HbA1c by about 1-2% (11) and in determining substantial weight loss (11, 51). They also improve the lipid panel, drastically reducing cardiovascular risk (52).

The main disadvantage of GLP-1 analogues is the frequent occurrence of gastrointestinal side effects, with nausea, vomiting and diarrhoea (51). However, these symptoms often occur only during the first days of therapy and are transient, and can be improved by slightly reducing the drug dose.

## Sulfonylureas

Sulfonylureas are among the first hypoglycaemic molecules used in T2DM. These drugs have both a direct action on pancreatic beta-cells and an extra-pancreatic action. Their hypoglycaemic effect occurs independently of blood glucose levels (53), and when they bind the pancreatic SUR1/Kir6.2 receptor they cause a closure of the potassium channels and depolarization of the cell membrane and thus the release of insulin (54). With a paracrine effect, sulfonylureas determine reduction of release of glucagon levels by pancreatic alpha cells, as a consequence of insulin secretion (55), and at a systemic level, a reduction in insulin resistance, in response to the improved glycaemic compensation (53). In monotherapy these drugs result in a 20-40 mg/dl decrease of blood glucose values and in a 1-2% decrease of HbA1c values (56).

The extra-pancreatic effects are various, from reduction of hepatic insulin clearance, to dose-dependent stimulation of glucose uptake, lipogenesis and glycogenesis.

The metabolism of almost all sulfonylureas is mostly hepatic and their elimination occurs by a renal route, exposing patients with hepatic, renal and cardiac insufficiency to a greater risk of hypoglycaemia, notably in malnutrition and debilitated and elderly conditions.

Another interesting finding is the higher rate of secondary failure obtained with these drugs compared to other hypoglycaemic ones, mostly due to the refractory reaction of beta cells in response to the chronic closure of potassium channels induced by sulphonylureas (53). Furthermore, in consideration of the high risk of hypoglycaemia and the direct effect that sulfonylureas exert on cardiomyocytes, the risk of cardiovascular events is very high in patients treated with these drugs (57). Therefore, in the last 10 years there has been a progressive reduction in their use in clinical practice in favour of new generation hypoglycaemic drugs with cardioprotective effects.

## Pioglitazone

Pioglitazone is a thiazolidinedione which acts as an exogenous agonist of peroxisome proliferator-activated receptor gamma (PPARγ). It is a hypoglycaemic drug with potent insulin-sensitizing action. In addition to reducing blood glucose and HbA1c values, and preserving beta cell function, pioglitazone has multiple beneficial effects both on the cardiovascular system and on the metabolic syndrome (58). Indeed, it improves the lipid profile by reducing triglycerides and free fatty acids, increasing HDL levels and making LDL less atherogenic. The antiatherogenic effect of pioglitazone is also expressed through activation of the PPARγ receptor, causing a direct anti-inflammatory and antioxidant effect on the arterial wall and reducing the risk of myocardial infarction (58–60).

However, there are some limitations in the use of pioglitazone, because it causes renal sodium retention and vasodilatation, resulting in oedema, which is more severe when pioglitazone is combined with sulfonylureas or insulin. For this reason, its use in patients with heart failure is not recommended, although it does not cause diastolic dysfunction or blood pressure increase (61). Pioglitazone can also induce weight gain, due to a water retention effect, as well as an increase in lean body mass that occurs in response to the increased appetite induced by direct stimulation of the hypothalamic PPARγ receptors (62). It is also associated with high risk of bone fractures, especially in post-menopausal women and after traumatic injury. Furthermore, the data concerning the increased risk of bladder cancer are controversial (58).

## Insulin

In patients with T2DM, the use of insulin therapy is indicated in the case of poor glycaemic control, secondary failure or contraindication to other hypoglycaemic agents. Insulin therapy can be combined with other oral agents or short-acting insulin (basal-bolus scheme), with the aim of mimicking the physiological pattern of insulin secretion. There are short-acting insulins, intermediate-acting insulins and slow-acting insulins. However, insulin therapy exposes patients to high risk of hypoglycaemia, and therefore requires adequate education for its management. In addition, it causes weight gain and has no proven effects on cardiovascular health (63, 64).

## Neuroendocrine tumours (glucagonoma and pheochromocytoma)

Neuroendocrine neoplasms are a rare group of lesions, mostly benign, formed by cells with ‘Neuro’ properties, which store monoamines, and ‘Endocrine’ ones, which synthetize them (65).

Some of them are characterized by hormonal hypersecretion with secondary comorbidities, including DM.

Glucagonoma originates from pancreas’ alpha-cells, and secretes high glucagon levels (66). The incidence is near to 2% of all pancreatic neuroendocrine neoplasms (pNENs), generally about 2 cases per million people per year (67, 68).

High glucagon levels (diagnostic when blood glucagon levels are >500pg/ml) (67) secreted by the tumour are associated with some specific and non-specific comorbidities including necrolytic migratory erythema (55-90%), weight loss (60-90%), DM or glucose intolerance (30-90%), mucosal lesion (glossitis, cheilitis, stomatitis) (30-40%), diarrhoea (10-15%), anaemia (30-80%), hypo aminoacidemia, low zinc levels, deep vein thrombosis (50%) and depression (50%) (66, 69, 70).

Glucagon is a hormone that stimulates hepatic gluconeogenesis and lipolysis and inhibits glycogenolysis to prevent hypoglycaemia. Furthermore, glucagon has been found to induce production of kisspeptin 1, a protein involved in suppression of insulin production (71, 72). These phenomena cause reduction of insulin secretion and increase in insulin resistance. Moreover, the degree of glucose increase has been shown to correlate with tumour size and metastasis (66).

Pheochromocytoma is a tumour that originates from adrenal medulla cells and commonly produces catecholamines (epinephrine, norepinephrine, and dopamine). On rare occasions, pheochromocytoma is biochemically silent (73). This tumour is rare, with an incidence of 2-8 per million people (74), and it is often associated with arterial hypertension and DM.

In our body there are two adrenergic receptors (α2 and β2) that are expressed on different cells and regulate glucose homeostasis. Particularly, stimulation of α2 receptors on pancreatic β-cells inhibits insulin secretion. In addition, stimulation of α2 and β2 receptors on pancreatic α-cells results in glucagon level increase and gluconeogenesis. Similarly, stimulation of hepatic α2 and β2 receptors results in glucagon hormone increase (75, 76). Catecholamines may induce insulin resistance through desensitization of β-adrenergic receptors. Moreover, stimulation of β3 and α1 receptors increases lipolysis and activation of β2 and α1 determines intake of glucose in myocytes (77). Explaining reduction of glucose uptake is quite tough. Han and Bonen reported that both insulin and epinephrine increase GLUT4 expression on the plasma membrane, but when their concentration is high at the same time, epinephrine reduces the carrying capacity of GLUT4, inducing insulin resistance (78).

### Treatment of secondary diabetes mellitus

**Figure 1:**
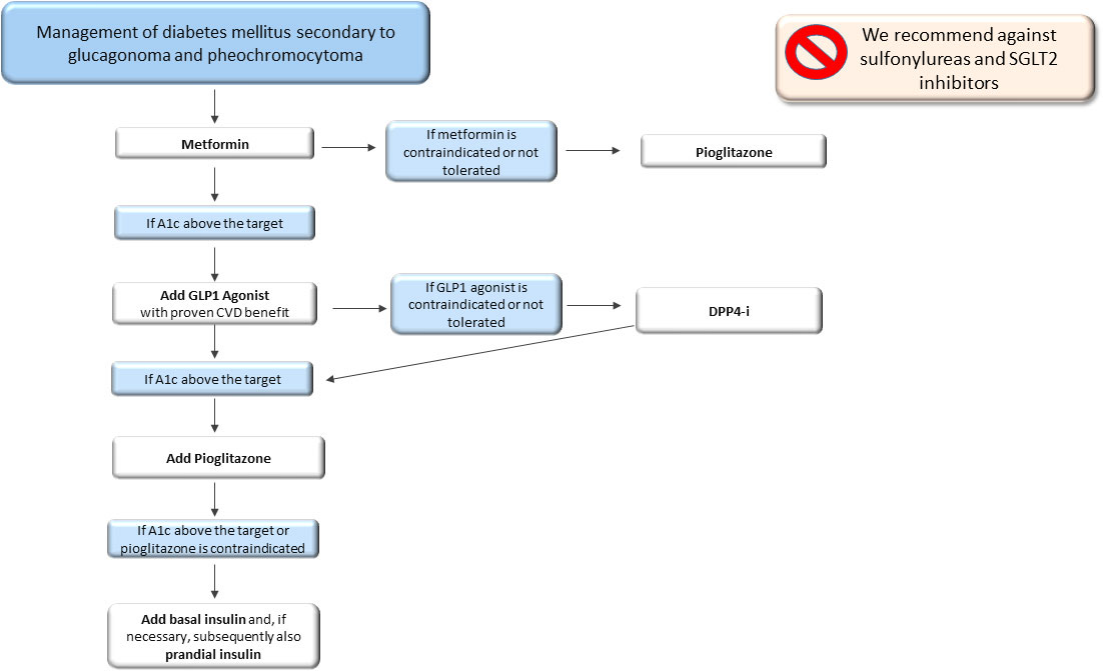
Therapeutic algorithm of diabetes mellitus secondary to glucagonoma and pheochromocytoma. A1c, glycated haemoglobin; CVD, cardiovascular disease; DPP4-i, Dipeptidyl peptidase inhibitors.

Surgery of the neuroendocrine tumours mentioned above is the best treatment to solve T2DM (79). However, until surgery is performed, metformin should be used as the first-line therapy, improving insulin sensitivity. Unfortunately, the clinical findings about hyperglycaemia treatment in patients with pheochromocytoma are scarce, and only a few case reports show a good glycaemic control obtained with metformin or insulin (75). GLP1-RA can also represent a good treatment option. Indeed, hypersecretion of catecholamines induces suppression of glucoregulatory and gut hormones, including GLP-1, contributing to impairing glucose metabolism (77, 80, 81). In addition, GLP1-RA could be very useful for their protective cardiovascular effects, considering that patients with neuroendocrine tumours are at high risk of cardiovascular complications. In patients with contraindications to GLP1-RA, DPP4-i could be used. When using GLP1-RA or DPP4-i alone or combined with metformin is not enough to adequately manage hyperglycaemia, we suggest adding pioglitazone. Metformin and pioglitazone, in addition to their metabolic effect, also have an inhibiting effect on pheochromocytomas cell proliferation (82, 83). As a last step, basal insulin or a basal bolus insulin scheme should be used when glycaemic control is difficult to obtain. We suggest not using sulfonylureas due to the absence of cardioprotective effects and we recommend against SGLT2-i due to their activity on the Na/K transporter, which has stimulatory effects on glucagon secretion (**Figure 1**).

## Cushing’s syndrome

Cushing’s syndrome (CS) is the consequence of an exogenous or endogenous excess of glucocorticoids (GCs), resulting from an adrenal or pituitary tumour or more rarely from a neuroendocrine ACTH secreting tumour (84).

CS annually affects about 0.2-5.0 per million people, with a female preponderance, notably in the pituitary form (84). The female-to-male ratio ranges from 3 to 5: 1 (84, 85) for pituitary ACTH secreting tumours, while by contrast there is a male-to-female ratio of 3.1 for neuroendocrine ACTH secreting tumours.

Patients with CS show phenotypical characteristics including moon face, facial plethora, striae rubrae, supraclavicular and dorsal fat pads (86) and several comorbidities including DM, visceral obesity dyslipidaemia, cardiovascular disease, osteoporosis and vertebral fractures, infections and neuropsychiatric disorders, which can sometimes persist even after the disease is cured and contribute to increased mortality, compared to the general population (87, 88).

Up to 50% of CS patients have impaired glucose metabolism to variable degrees. In some patients, GC excess is associated with higher postprandial glucose levels with normal fasting serum glucose. However, the oral glucose tolerance test should be performed in all patients with CS to accurately detect glucose metabolism disorders (89).

It is estimated that about 20-47% of CS patients have overt DM and 21-64% have impaired glucose tolerance. The development of glucose intolerance in CS is also greatly influenced by age, genetic predisposition and lifestyle factors, as recently reported (88, 90). Interestingly, there are no gender-specific variations in frequency (91).

The pathophysiology of GC-induced diabetes involves both insulin resistance and impaired insulin production. Insulin resistance is a common comorbidity of CS. GCs affect insulin secretion and action. GC excess triggers expression of several enzymes involved in gluconeogenesis, increasing glucose synthesis. Moreover, it reduces insulin sensitivity, both directly by interfering with the insulin receptor signalling pathway, and indirectly by promoting lipolysis and proteolysis (88).

Insulin binding to its own receptor results in phosphorylation of insulin receptor substrates (IRS) by phosphoinositide-3-kinase (PI3K). Then it activates protein kinase B, protein kinase C, and GLUT4 translocation to the membrane, resulting in glucose uptake. Expression and phosphorylation of AKT are both decreased by GCs, which results in a 40% reduction in glucose absorption. Moreover, GCs stimulate serum- and GC-inducible kinase 1 (SGK1), which raises the phosphorylation of the forkhead box protein O1 (FOXO1) and lowers the phosphorylation of protein kinase B in adipocytes, resulting in insulin resistance (92).

Glucocorticoids cause post-receptor defects in the insulin receptor substrate-1 (IRS-1), phosphatidylinositol-3 kinase (PI3K), and protein kinase B resulting in anti-insulin effects in the liver, skeletal muscle, and adipose tissue. As a result of these activities, glucose transporter migration to the cell surface is inhibited, reducing glucose absorption. Additionally, GCs inhibit glycogen synthesis in skeletal muscle by reducing phosphorylation of insulin-stimulated glycogen synthase kinase-3 (93).

GCs could affect release of adipokines like adiponectin and leptin, which might have a significant effect on insulin sensitivity (94). The effects of hypercortisolism in skeletal muscle include decreased insulin sensitivity, reduced glucose absorption, and inhibited glycogen synthesis (95). Moreover, insulin resistance is more likely to develop in CS due to body fat distribution, increased visceral adiposity, and subsequent onset of metabolic syndrome (96).

In addition to their effects on insulin sensitivity, GCs also inhibit insulin secretion from pancreatic b-cells (97). The impact is also influenced by the dose of GCs and the duration of exposure. Studies conducted *in vitro* reveal a direct suppression of insulin secretion, which may be brought on by a reduction in the transcription of genes necessary to initiate the secretory process in response to cytoplasmic calcium levels (98). The compensatory beta cell hyperplasia and hyperinsulinaemia brought on by this GC-induced insulin resistance can lead to normoglycaemia.

In addition, persistent GC exposure also has an impact on other pancreatic islet cells. Treatment with GCs results in higher glucagon levels and alpha cell mass. High levels of glucagon cause hyperglycaemia because they accelerate gluconeogenesis and glycogenolysis and inhibit glycolysis and glycogen synthesis (92). Furthermore, short-term GC exposure reduces the insulinotropic effects of GLP1 (99).

### Impact of CD medications inglucose homeostasis

Surgery represents the first-line therapy for CD. Generally, normalization of hypercortisolism is associated with improvement of glucose metabolism, even though in some cases it may persist (100). CS medications and antidiabetic treatment can be used as combined treatment in those patients for whom surgery is contraindicated or in the meantime to undergo it.

CD medications affect glucose homeostasis independently of the pharmacological regulation of hypercortisolism.

The somatostatin analogue pasireotide (SOM230) has a substantially greater affinity for somatostatin receptor 5 (SSTR5) than other somatostatin analogues. Since SSTR5 is expressed on ACTH-secreting adenomas and is not downregulated by GC excess, it is the perfect target for pasireotide.

Pasireotide causes frequent hyperglycaemic events because the SSTR5 is expressed by pancreatic ß-cells, which in turn control insulin production. As a result, patients treated with pasireotide show decreased insulin secretion. The clinical importance of this effect is compounded by the persistence of insulin resistance caused by hypercortisolism even when treated with pasireotide. About 5.6% of patients treated with pasireotide were reported to have stopped it due to the development of hyperglycaemic events (101). In addition, in the phase 2 and 3 clinical trials, 41% and 50% of patients treated with pasireotide, respectively, started an antidiabetic treatment (102, 103).

Cabergoline treatment is associated with improvement of DM and glucose intolerance by 60% and 46%, respectively, independently of the control of hypercortisolism, due to a direct effect of this drug (104). Similarly, bromocriptine, another dopaminergic drug, has been shown to restore normal glucose homeostasis in patients with DM, most likely by boosting insulin-mediated inhibition of hepatic glucose synthesis or improving splanchnic glucose absorption (105).

Other cortisol-lowering drugs include adrenal-directed drugs. Ketoconazole, an inhibitor of cytochrome P450-dependent enzymes, has been shown to enhance glucose metabolism in individuals with DM and hypercortisolism at doses ranging from 200 to 1000 mg/day (106). Levoketoconazole, a 2S, 4R enantiomer of ketoconazole, has been shown to improve glucose metabolism after normalization of hypercortisolism, with a decrease in HbA1c and fasting plasma glucose (64, 107).

Metyrapone, an inhibitor of adrenal 11β-hydroxylase, has been reported to improve glucose metabolism in 80% of patients with CS, as a secondary effect of the biochemical control of hypercortisolism (108, 109). Osilodrostat, a potent inhibitor of 11β-hydroxylase and aldosterone synthase showed a significant decrease in glucose levels during treatment, parallel to the decrease in urinary free cortisol levels (110).

Mifepristone, a glucocorticoid receptor antagonist, was also reported to be associated with improved insulin sensitivity in 60% of patients with DM or glucose intolerance (111). Similarly, relacorilant, a selective glucocorticoid receptor modulator, has been shown to be associated with improvement of glucose metabolism (112).

### Treatment of secondary and pasireotide-induced diabetes mellitus

Treatment of secondary DM in patients with CS can take advantage of biochemical control of hypercortisolism, although in many cases control of hypercortisolism does not completely result in diabetes resolution. Currently, there is no evidence of the ideal antidiabetic therapy for CD, due to the scarcity of patient-based studies. Indeed, the current recommendations for treatment of DM in CD are based on expert opinions and general algorithms for treatment of T2DM (113–116).

Due to the insulin resistance condition, metformin should be used as a first-line therapy combined with GLP1-RA, which show cardiovascular protective effects and intermediate to very high weight loss. Indeed, patients with CD show high cardiovascular risk due to the presence of many cardiovascular risk factors such as hyperglycaemia and dyslipidaemia, but also endothelial dysfunction and chronic inflammation and for this reason cardio-protection is strongly required (117). In addition, a decreased GLP-1 function in CD was recently reported, supporting the usefulness of treatment with GLP1-RA (118).

In patients with contraindications or non-toleration of GLP1-RA, DPP4-i could be used, even though no clinical evidence in CD patients has been reported. With regard to SGLT2-i, despite their effect in reducing insulin resistance and protecting the cardiovascular system, they are associated with a high risk of genitourinary mycotic infections and for this reason they are not strongly recommended and should be used with caution, notably in patients with severe hypercortisolism.

**Figure 2:**
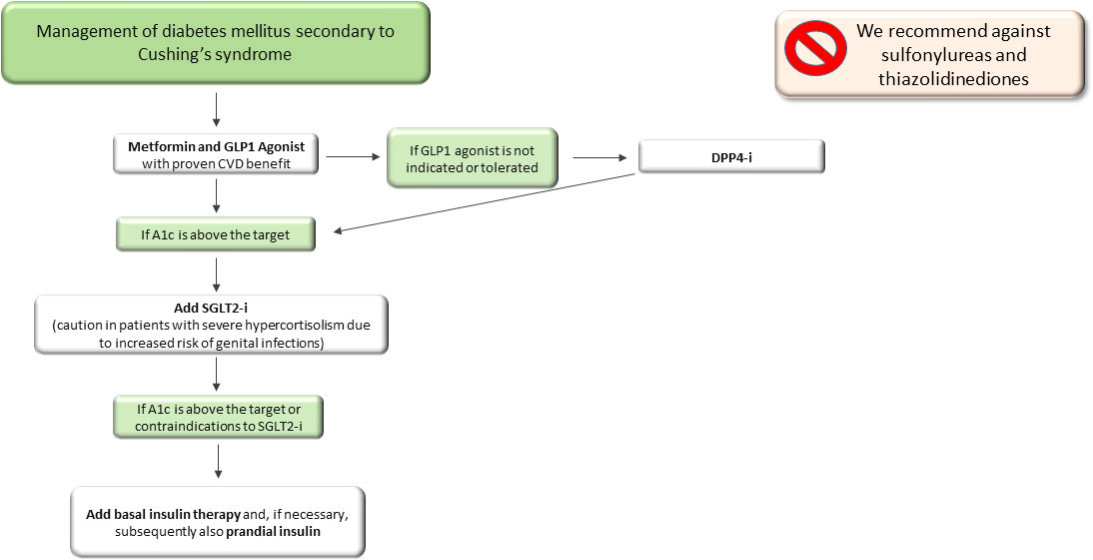
Therapeutic algorithm of diabetes mellitus secondary to Cushing’s syndrome. A1c, glycated haemoglobin; DPP4-i, Dipeptidyl peptidase inhibitors; SGLT2-i, sodio-glucose co-transporters 2 inhibitors.

For patients who do not adequately achieve good glycaemic control or show contraindication to the above-mentioned drugs, insulin treatment should be suggested, first as long-acting insulin and after as a basal-bolus scheme (**Figure 2**). We recommend against the use of sulfonylureas, due to weight gain and lack of effect on cardiovascular disease, and thiazolidinediones, due to increased bone fracture risk, already very high in patients with CS, and fluid retention, even though a successful decrease in HbA1c levels was reported in a patient treated with pioglitazone (119).

In patients with pasireotide-related hyperglycaemia a significant decrease in HbA1c and fasting plasma glycaemia was obtained using GLP1-RA compared to other antidiabetic drugs such as metformin and DPP4-i (120). Further, a case of a man with pasireotide-induced hyperglycaemia successfully treated with a combination of GLP1-RA, insulin and empaglifozin was also reported (121). The most robust findings are those reported by Samson et al., who showed the superiority of the combination of sitagliptin and liraglutide in achieving glucose control (122).

Trementino et al. reported a case of a 55-year-old woman treated with insulin and metformin with achievement of good glucose control (123).

**Figure 3:**
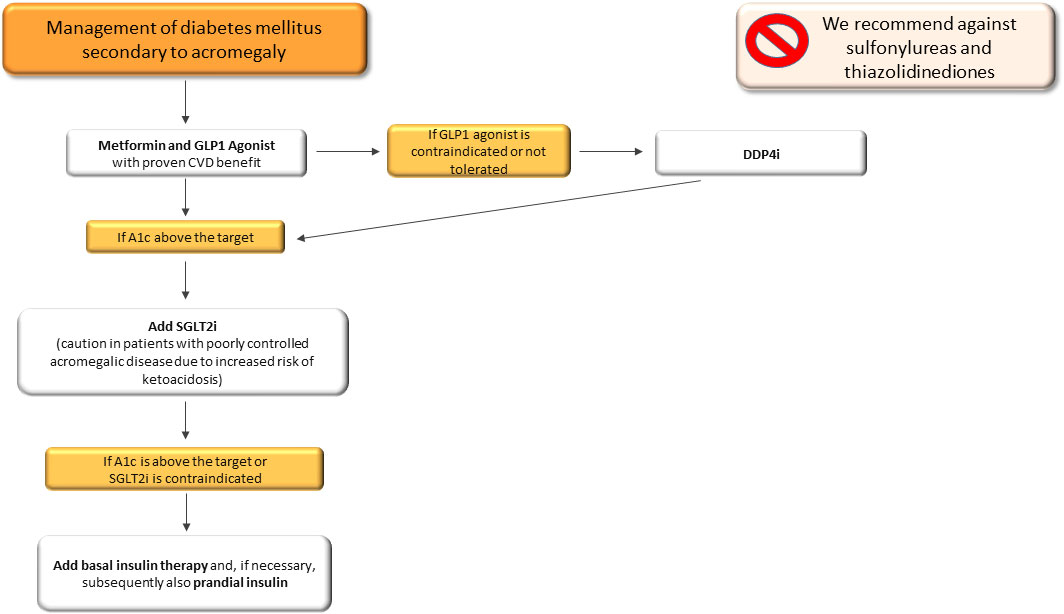
Therapeutic algorithm of pasireotide-related hyperglycaemia in patients with Cushing’s syndrome and acromegaly. CVD, cardiovascular disease; A1c, glycated haemoglobin; SGLT2-i, sodio-glucose co-transporters 2 inhibitors.

In summary, in patients with pasireotide-related hyperglycaemia we suggest starting GLP1-RA and metformin as a first-line approach and avoiding DPP4-i, in accordance with the findings of the previous studies (120, 121) and to the mild anti-hyperglycaemic effects of DPP4-i (**Figure 3**).

## Acromegaly

Acromegaly is characterized by GH and consequently IGF-1 hypersecretion, which occurs in adults, after conjugative cartilage welding. It can be caused by a GH-secreting adenoma. It is therefore distinguished from gigantism, a condition in which GH hypersecretion is observed in childhood or puberty, and characterized by a different spectrum of clinical alterations, mainly by an increase in height generally more than two standard deviations from the subject’s genetic target. Only about 5% of cases of acromegaly are due to GH hyperproduction or ectopic GHRH-secreting tumour (pancreatic insular tumours, lung microcitomas, breast tumours, ovarian tumours, gliomas, hypothalamic gangliocytomas). Generally, acromegaly arises sporadically; more rarely it may be observed within various syndromes such as neurofibromatosis type 1, McCune-Albright syndrome, MEN1 and MEN4 (124). It shows an estimated prevalence ranging from 2.8 to 13.7 cases per 100,000 subjects and an annual incidence between 0.2 and 1.1 cases per 100,000 (125), higher than that previously reported (126, 127). Data on familial forms of acromegaly associated with MEN1 are very limited, with an overall prevalence rate <1% in acromegalic subjects (128, 129). The average age at diagnosis is about 50 years, believed to be with an average diagnostic delay of about 5 years. There are no significant differences between genders, although some studies based on individual geographic populations suggest a higher prevalence in female subjects (130) and a lower age at diagnosis in male subjects (131).

The clinical manifestations of acromegaly are due to an excess of GH and IGF-1. In patients in whom no conjugate cartilage welding has occurred, cartilage proliferation and elongation of the long bones are observed, resulting in stature increase and gigantism. On the other hand, adults show the typical acromegalic facies, due to periosteal bone tissue and increased diameter of the long bones. The deformities are most evident in the acral regions and include protrusion of the frontal bosses and zygomatic arches, enlargement of the nasal pyramid, lips and tongue, dental diastasis and skin thickening. The appearance of these alterations is gradual and is one of the main reasons for diagnostic delay. The above-mentioned bone changes are often accompanied over time by arthritic manifestations, functional activity disorders and pain. Sexual disorders, oligo-amenorrhoea and hirsutism in female subjects can also be observed in cases of mixed GH-PRL-secreting adenoma.

The most frequent comorbidities include arterial hypertension, DM, visceromegaly, cardiovascular and pulmonary complications. Diabetes mellitus is the most frequent metabolic complication of this disease. Over the years, the role of cardiovascular complications and their effect on disease prognosis, especially arterial hypertension, have been investigated (132, 133). However, more recent data support the role of cancer as the main factor in acromegaly-related mortality (134, 135). Metabolic comorbidities mostly influence the long-term risks related to acromegaly, and thus its mortality. Notably, DM is the most frequent metabolic complication in acromegalic patients, and can often persist even after multimodal treatment (136).

The most frequent metabolic complications of acromegaly are impaired glucose tolerance and DM, which can be already present at the time of diagnosis in approximately 30-50% of cases (137). The pathophysiological link between diabetes and acromegaly is given by the association between elevated GH levels and insulin resistance, which especially in patients with a long history of the disease can result over time in impaired glucose tolerance to the point of insulin deficiency and diabetes (138). High GH levels affect not only lipid metabolism, but also fatty acid metabolism. GH promotes lipolysis, which leads in acromegalic subjects to a reduction in fat accumulation, a characteristic feature of acromegaly (139). Lipolysis is one of the main pathophysiological mechanisms underlying insulin resistance in acromegalic patients. In particular, fatty acids reduce glucose utilisation in peripheral tissues and promote insulin resistance and hyperglycaemia (140). Acromegaly is the only disease with simultaneous elevation of circulating GH, IGF1 and insulin levels, and in which insulin resistance is associated with a reduction in body fat deposits, especially liver fat (141). In healthy subjects, fatty acids inhibit GH release, whereas in acromegalic patients there is a loss of this feedback (142). Over time these effects cause lipotoxicity and beta-cell deterioration (143). These effects seem to be resolved in surgically treated patients, where a normalisation of this metabolic shift is observed (144).

Screening for DM should be recommended in all patients at diagnosis of acromegaly, especially in younger patients.

### Impact of acromegaly medications in glucose homeostasis

In patients who have contraindications to surgery or refuse surgery, medical therapy is recommended, including dopamine agonists, somatostatin analogues and pegvisomant.

In patients with poor response to first-generation somatostatin analogues, pasireotide may be used. Recently, it was shown that somatostatin analogues impair insulin, glucagon and incretin secretions (145).

As mentioned for CS, cabergoline is associated with a reduction in both fasting blood glucose and HbA1c (146). These beneficial effects are indirectly mediated by the action on the dopaminergic and sympathetic systems since these drugs have no specific receptors active on metabolism.

Recent evidence supports the positive effects on glycaemic control of pegvisomant, the only GH-receptor-agonist currently available, which has shown a favourable effect on the reduction of glycaemic values, as demonstrated by an analysis of the data obtained from the ACROSTUDY (147, 148), with more significant effects on glycaemic control observed than those induced by octreotide (149). Pegvisomant is also associated with improved peripheral insulin sensitivity (150) and combination with somatostatin analogues was associated with improvement of GLP1 secretion (145).

### Treatment of secondary and pasireotide-induced diabetes mellitus

Treatment of secondary diabetes in patients with acromegaly benefits from treatment of acromegaly itself. Surgery, which represents the first-line treatment for acromegaly, is generally associated with diabetes, glucose tolerance defects and insulin-resistance resolution (130–132, 151–153). However, surgery is not always associated with acromegaly remission, which in some cases may persist, despite acromegaly biochemical control (136).

Current therapeutic management of diabetes secondary to acromegaly is based on expert opinions and general recommendations of typical T2DM (114, 154).

Clinical studies on treatment of diabetes in acromegaly have shown that a large percentage of patients achieved good glycaemic control with metformin in monotherapy or combined with other oral antidiabetic drugs (155). In addition, a minority of them were insulin-treated with a good glucose control (155).

Pioglitazone has also been successfully used as antidiabetic treatment in patients with acromegaly, as shown in two case reports (156). Preclinical findings showed that thiazolidinediones also reduced circulating GH and IGF-1 levels and GH pituitary cell hormone secretion (157, 158). However, clinical experience did not confirm the efficacy of pioglitazone in obtaining biochemical control of acromegaly (159).

SGLT2-i were also used in patients with DM secondary to acromegaly, showing a significant decrease in HbA1c levels (160). However, a patient who developed ketoacidosis during treatment with SGLT2-i was reported (161), and due to the tendency of these drugs to favour such adverse events, Zaina et al. suggested using SGLT2-i only in patients who had biochemically controlled acromegaly, had undergone surgery or were pharmacologically treated, taking caution in those patients with uncontrolled disease and at high risk of developing diabetic ketoacidosis (162).

With regard to pasireotide-related hyperglycaemia, some studies reported interesting results. Pasireotide, as mentioned above, is associated with reduced levels of circulating insulin and incretin hormones (163). Not all patients treated with pasireotide require pharmacological intervention for glycaemic control (164). Indeed, in some cases only a lifestyle modification was associated with good glucose control, and when antidiabetic treatment was required, metformin alone was successful in achieving glucose control (165).

A recent multicentric study compared the efficacy of the combination of DPP4-i (sitagliptin) and GLP1-RA (liraglutide) vs. insulin treatment, as second-line therapy after metformin, showing the superiority of the first arm of treatment (122). However, another study showed that use of DPP4-i was associated with an increase in GH secretion, and therefore these drugs need to be used with caution (166).

The therapeutic choice of GLP1-RAs is not only more effective on glycometabolic control but also counteracts the reducing effect of endogenous incretins brought about by pasireotide (120), which does not so much act on peripheral insulin sensitivity but worsens glycometabolic compensation by reducing insulin and incretin hormone levels (163).

A recent study by Gadelha et al. showed positive effects in counteracting pasireotide-related hyperglycaemia by metformin alone or in combination with other antidiabetic drugs, achieving the HbA1c goal <7% (167).

**Figure 4:**
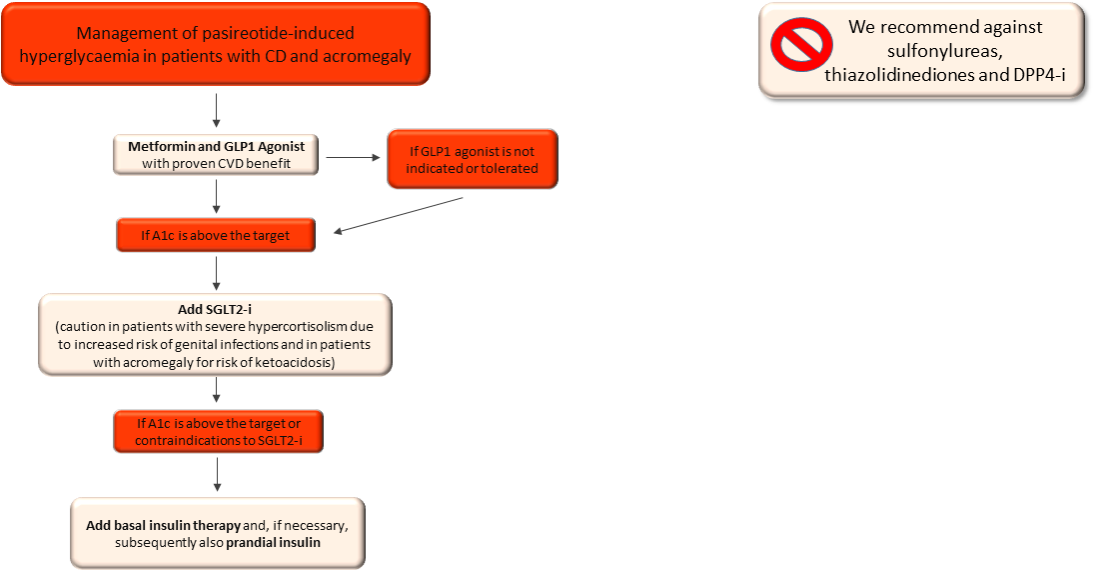
Therapeutic algorithm of diabetes mellitus secondary to acromegaly. A1c, glycated haemoglobin; CVD, cardiovascular disease; SGLT2-i, sodio-glucose co-transporters 2 inhibitors; DPP4-i, Dipeptidyl peptidase inhibitors.

In summary, therapeutic management of secondary DM in acromegalic patients should be based on a combination of metformin and GLP1-RA, as a first therapeutic approach, in line with the latest scientific evidence, in order to take advantage of the cardioprotective effects of GLP1-RA. In patients with contraindications or non-toleration of GLP1-RA, DPP4-i should be taken into account. Particular caution should be paid to the use of SGLT-2 due to the increased risk of developing ketoacidosis (161). In addition, we recommend against sulfonylureas due to their effect on weight gain and the absence of cardiovascular benefits and thiazolidinediones due to their fluid retention effect, with a potential negative effect on acromegalic cardiac disease (**Figure 4**). With regard to pasireotide-induced DM, we suggest a similar therapeutic algorithm to that of secondary DM (**Figure 4**), avoiding the use of DPP4-i, due to their mild effects on hyperglycaemia (**Figure 4**).

## Conclusions

Diabetes mellitus secondary to endocrinopathies is a challenge for endocrinologists. It can be caused by an excess of counterregulatory hormones able to induce insulin resistance and glucose metabolism disorders at variable degrees. As also recommended by the ADA guidelines, metformin alone or combined with GLP-1 RA represents the first line therapy, mainly due to the effect on improving insulin resistance, weight loss and cardiovascular protective effects. When GLP-1 RA are contraindicated or not tolerated, DPP4-i should be used. In patients with pheochromocytoma and glucagonoma, pioglitazone could be used as the third-line therapy, and when glycaemic control is not obtained, insulin therapy should be started. By contrast, in patients with acromegaly and CS we recommend not using sulfonylureas and thiazolidinediones and we suggest using metformin combined with GLP-1 RA as the first-line therapy, and in cases of inadequate glycaemic control, SGLT2i could be indicated. Surgery is generally associated with a resolution of the disease and an improvement of glucose metabolism. For this reason, target therapies associated with antidiabetic medications can be used as adequate strategies for control of glycaemic values.

In pasireotide-related hyperglycaemia the combination of metformin and GLP1-RA is strongly recommended, and in cases of poor glycaemic control insulin treatment should be used.

Larger prospective studies on the treatment of diabetes secondary to counterregulatory hormone excess syndromes are required in order to further support our clinical real-life based experience.

## Author contributions

Conceptualization, VG and CG. Writing—original draft preparation, FE, RS, CA, MB, MM. Writing—review and editing, VG and CG. SUPERVISION, CG. Project administration, CG. All authors contributed to the article and approved the submitted version.
